# Effects and interactive effects of high-altitude environment on metabolism in normal and diabetic populations: a comparative metabolomics study

**DOI:** 10.3389/fendo.2026.1736270

**Published:** 2026-02-12

**Authors:** Jing Shi, Jinli Meng, Chenghui Zhang, Li Feng, Suyuan Wang, Hengyan Li, Yongyue Guo, Xin Hu, Xiaomei Li, Wanlin He, Jian Cheng, Yunhong Wu

**Affiliations:** 1 Science and Education Section, Hospital of Chengdu Office of People’s Government of Tibetan Autonomous Region (Hospital C.T.), Chengdu, Sichuan, China; 2 Department of Radiology, Hospital of Chengdu Office of People’s Government of Tibetan Autonomous Region (Hospital C.T.), Chengdu, Sichuan, China; 3 Department of Endocrinology and Metabolism, Hospital of Chengdu Office of People’s Government of Tibetan Autonomous Region (Hospital C.T.), Chengdu, Sichuan, China; 4 Department of Neurosurgery, West China Hospital, Sichuan University, Chengdu, China

**Keywords:** biomarker, guanosine, high altitude, metabolomics, type 2 diabetes mellitus

## Abstract

**Introduction:**

High-altitude environments impose unique physiological stresses that may alter metabolic dysregulation in individuals with type 2 diabetes mellitus (T2DM). However, the key determinants driving altitude-specific metabolic differences in diabetic patients remain insufficiently characterized. This study aimed to identify critical metabolic biomarkers and pathways distinguishing T2DM patients residing at high versus low altitudes.

**Methods:**

Serum samples were collected from 100 participants stratified into four matched groups: high-altitude T2DM (H_T2DM), high-altitude healthy controls (H_HC), low-altitude T2DM (L_T2DM), and low-altitude healthy controls (L_HC). Metabolomic profiling was performed using ultra-performance liquid chromatography‒quadrupole time-of-flight mass spectrometry (UPLC‒Q‒TOF‒MS) to compare endogenous metabolite abundance across groups. Pathway topology analysis was conducted to annotate functional metabolic pathways, and multicenter validation was implemented to verify the robustness of candidate biomarkers.

**Results:**

A total of 26 differentially abundant endogenous metabolites were identified in H_T2DM patients relative to the other three groups, with 18 metabolites significantly upregulated and 8 downregulated. Pipecolic acid, lauric acid, guanosine, and kaempferol were identified as potential early biomarkers for high-altitude diabetes, collectively achieving a prediction accuracy of 92%. These biomarkers were linked to core metabolic pathways including lysine degradation, fatty acid biosynthesis, and purine metabolism. Multicenter validation further confirmed guanosine as the most robust and altitude-specific biomarker for T2DM.

**Discussion:**

Our comprehensive metabolomic analysis reveals distinct metabolic perturbations in T2DM patients under high-altitude conditions, highlighting guanosine as a unique biomarker for identifying altitude-related diabetic metabolic dysregulation. These findings advance our understanding of the pathophysiological mechanisms underlying T2DM in high-altitude environments and provide a potential diagnostic target for clinical management of diabetic populations in such regions.

## Introduction

Diabetes is a chronic metabolic disease, and its incidence continues to rise worldwide. It has been reported that there are 463 million people with diabetes mellitus (DM) worldwide and the number is expected to reach 700 million by 2045 (1). Among these, type 2 diabetes accounts for nearly 90% and the national prevalence of type 2 diabetes mellitus (T2DM) is estimated to be 7.0% (5.3–8.7%), endangering global public health (2).

Surprisingly, various studies have shown a low prevalence of T2DM in high-altitude settlers, with the prevalence decreasing as altitude increases (3). A survey has revealed that the prevalence of diabetes on the Tibetan Plateau (7.5%) is much lower than its average prevalence in China (11.9%) (4). A cross-sectional study of U.S. adults revealed that living at a high altitude (3,500 m) was associated with a lower risk of diabetes than living at a low altitude (500 m) (5). It has also been reported that women residing in high-altitude environments have lower blood glucose concentrations before and during pregnancy than women residing in low-altitude environments (6). In addition, studies have shown that living at high altitudes in the Saudi Arabia can decrease blood glucose concentration (6). Thus, a high-altitude environment may alleviate diabetes symptoms compared with a low-altitude environment.

Diabetes is a metabolic disease. At present, there are few related studies on the effects of living at high and low altitudes on the metabolism of diabetic patients.

Metabolomics is an important area of systems biology that investigates changes in metabolites or biological systems over time or after stimulation or disturbance. By identifying differentially metabolites (DMs) between experimental and control groups the biological processes in which these DMs are involved are revealed, indicating their mechanisms of physiological activity (7). LC–MS/MS has been widely used in untargeted metabolomics research because it enables the analysis of a wide range of substances and involves relatively simple sample preparation (8).

**Figure 1:**
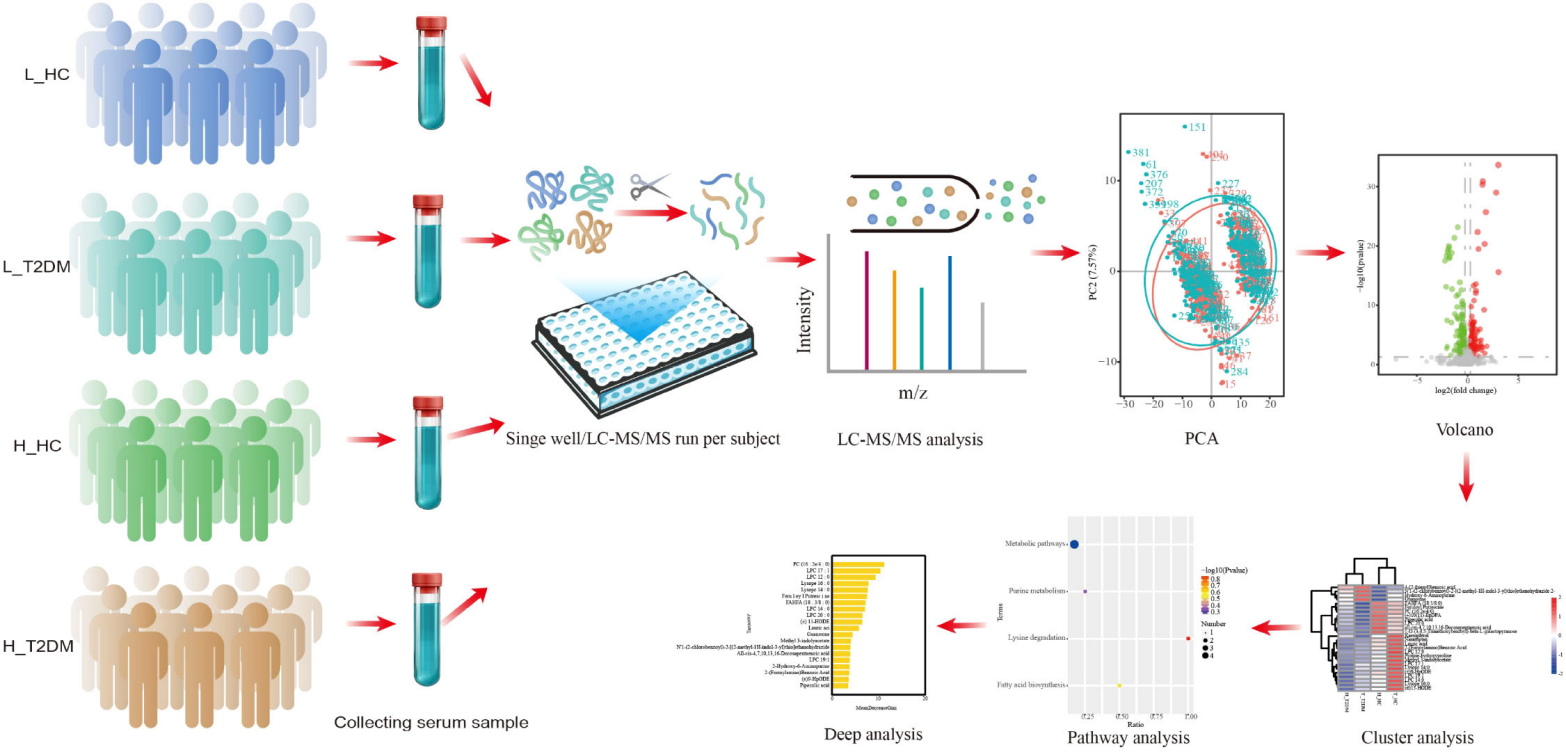
Overview of the serum metabolomics workflow. Samples from Tibetan and low-altitude diabetic patients and healthy controls were prepared in 96-well plates by an automatic liquid handling system and analyzed by LC–MS/MS.

We recruited 400 subjects and separated them into four groups: T2DM patients living at high altitudes (3500 m, H_T2DM), T2DM patients living at low altitudes (L_T2DM), healthy controls living at high altitudes (3500 m, H_HC), and healthy controls living at low altitudes (L_HC). Four hundred serum samples were obtained from these subjects. Liquid chromatography–tandem mass spectrometry (LC–MS/MS), analysis of covariance (ANCOVA) and machine learning were used to determine the potential determinants of diabetes between high and low altitude patients (**Figure 1**).

## Methods

### Study design and ethics approval

This cross-sectional study was conducted between October 31, 2022, and October 30, 2023, at the Hospital of the Chengdu Office of the People’s Government of the Tibetan Autonomous Region (Hospital C.T.). The protocol was approved by the Ethics Committee of Hospital C.T. (Approval No.: [(2024) No.41 Scientific Research] postpone). All participants provided written informed consent prior to enrollment.

### Study population

#### Eligibility criteria

Participants were stratified into four groups: high-altitude (Extended at ≥3,500 m) T2DM (H_T2DM), high-altitude healthy controls (H_HC), low-altitude (Extended at ≤ 500 m) T2DM (L_T2DM), and low-altitude healthy controls (L_HC).

Inclusion criteria: (1) Aged 20–65 years. (2) Newly diagnosed type 2 diabetes mellitus (T2DM) confirmed by oral glucose tolerance test (OGTT) in accordance with the guidelines of the American Diabetes Association (ADA) (fasting plasma glucose [FPG] ≥ 6.1 mmol/L or 2-hour post-75g glucose ≥ 7.8 mmol/L), without prior use of hypoglycemic medications. (3) Healthy controls (HCs) with normal OGTT results (FPG <6.1 mmol/L and 2-h post-load glucose <7.8 mmol/L) and no history of chronic diseases. (4) Altitude classification: Extended residence (≥5 consecutive years) at ≥3,500 m (high-altitude) or ≤500 m (low-altitude), verified via household registration records and utility bills.

Exclusion criteria: (1)Serious comorbidities (e.g., cirrhosis, heart failure, cancer); (2) Acute diabetes complications (e.g., diabetic ketosis, hyperglycemic hyperosmotic state) or advanced chronic complications defined as: stage 4–5 chronic kidney disease (urinary albumin-to-creatinine ratio ≥300 mg/g or estimated glomerular filtration rate <30 mL/min/1.73m²), active diabetic foot ulcers, or severe diabetic retinopathy (proliferative stage); (3) Family history of diabetes; (4) Recent use of hypoglycemic drugs or interventions; (5) Altitude relocation within 1 year.

#### Recruitment and enrollment

All T2DM participants who met the inclusion criteria were immediately subjected to sample collection. After sampling, all samples were uniformly subjected to clinical biochemical tests.

### Sample collection and processing

Fasting venous blood (10 mL) and urine samples were collected in the morning. Blood samples were processed within 2 hours of collection via standardized centrifugation (3,000g, 10 min, 4°C). Hemolyzed specimens (free hemoglobin >0.2 g/L, detected spectrophotometrically at 414 nm) were excluded. Aliquots of plasma and urine were stored at -80°C in the Hospital C.T. Biobank for metabolomics analysis. Residual samples were archived for future studies on diabetes complications (e.g., metabolite, protein, or gene sequencing assays), with de-identification via unique study IDs to protect privacy.

### Data collection and quality control

Clinical data: Demographics, medical history, and medication use were collected via EHR review and standardized questionnaires.

Follow-up: Endocrinologists and diabetes nurses conducted regular phone follow-ups to monitor blood glucose, blood pressure, and medication adherence, providing online interpretation of test reports and management guidance.

Confidentiality: Personal identifiers were replaced with codes; data access was restricted to the research team, with only de-identified data used for analysis and publication.

### Metabolomics analysis

#### Metabolite extraction

The serum samples (50 μl) were resuspended in prechilled 80% methanol by vortexing. The samples were incubated on ice for 5 min and centrifuged at 15000 × g and 4°C for 15 min. Some of the supernatant was diluted to a final concentration of 53% methanol with liquid chromatography–mass spectrometry (LC–MS)-grade water. The samples were subsequently transferred to a fresh Eppendorf tube and then centrifuged at 15000 × g and 4°C for 15 min. Finally, the supernatant was injected into the LC–MS/MS system for analysis.

#### LC–MS/MS analysis

LC–MS/MS analyses were performed using a Vanquish UHPLC system (Thermo Fisher, Germany) coupled with an Orbitrap Q Exactive™ HF-X mass spectrometer (Thermo Fisher, Germany) at Novogene Co., Ltd. (Beijing, China). Chromatographic conditions: The separation was performed on an ultra-high-performance liquid chromatography (UHPLC) column (100 mm length, 1.7-2.1 μm particle size) with a column temperature maintained at approximately 40°C and a flow rate of 0.3-0.4 mL/min. Fragmentation mode: High-energy collision-induced dissociation (HCD) was employed for MS/MS analysis, with collision energies dynamically adjusted based on the m/z of the parent ions (e.g., 20-40 eV). Metabolite identification criteria: Metabolites were annotated using an in-house secondary spectral library (Version V1/V2), which incorporates reference standards with precise mass accuracy (<5 ppm error), retention time (RT), and MS/MS fragment ion profiles. Level 1 identification required simultaneous fulfilment of three criteria: Accurate mass match (± 5 ppm),Retention time deviation within ±0.2 min, and MS/MS fragment match (≥80% similarity).

#### Data processing and metabolite identification

The raw data files generated with LC–MS/MS were processed using Compound Discoverer 3.1 (CD3.1, Thermo Fisher) to perform peak alignment, peak selection, and quantitation for each metabolite. The peaks were subsequently compared against the mzCloud (https://www.mzcloud.org/), mzVault and mass list databases to obtain accurate qualitative and relative quantitative results. Statistical analyses were performed using the statistical software R (version 3.4.3), Python (version 2.7.6) and CentOS (version 6.6).

We addressed the multiple comparisons issue using a combination of statistical thresholds and machine learning-based feature importance. Specifically, differentially expressed metabolites were identified by meeting all three criteria: 1) Variable Importance in Projection (VIP) score > 1 from orthogonal partial least squares discriminant analysis (OPLS-DA) model, 2) Fold change ≥ 1.2 (or ≤ 0.833) calculated by comparing group means, and 3) Adjusted p-value < 0.05 using Benjamini-Hochberg false discovery rate (FDR) correction on Student’s t-test results.

### Statistical analysis

IBM SPSS for Windows (version 22.0, Armonk, NY, USA) was used for statistical analysis and receiver operating characteristic (ROC) curve generation. All collected data are expressed as the mean ± standard deviation (SD). Differences between H_T2DM patients and L_T2DM patients were evaluated with Student’s t test followed by the Kolmogorov–Smirnov test. A *P* value less than 0.05 was considered to indicate statistical significance.

## Results

### Comparison of clinical data between H_T2DM patients and L_T2DM patients

In this study, we compared the clinical data of 100 H_T2DM patients and 100 L_T2DM patients. There were no significant differences between H_T2DM patients and L_T2DM patients in terms of sex distribution, body mass index (BMI), height, sex. However, the body weight of the H_T2DM group was significantly higher than that of the L_T2DM group (P = 0.040). This might be related to the high intake of animal fat, red meat and refined carbohydrates in the high-altitude area, combined with the lack of sufficient oxygen in the high-altitude region, which makes it impossible to engage in extensive physical activities. The average age in the H_T2DM group was younger than that in the L_T2DM group (P < 0.001), which reflects a significant change in the diabetes epidemic trend in the plateau region. This may be related to the dietary structure shift: the traditional high-fiber, low-fat diet (such as foods made from barley) is being replaced by modern diets rich in saturated fats, refined carbohydrates, and sugary beverages, which accelerates insulin resistance in the younger population. It may also be due to the differences in the spread of health education, where in low-altitude urban centers, young people pay more attention to health preservation, exercise, and can more conveniently access regular health check-ups, enabling early intervention and treatment. In contrast, the health education coverage rate is low in high-altitude areas, compounded by the altitude factor, limited aerobic exercise, and limited medical resources, making it impossible to receive timely diagnosis and treatment. This results in a lower average age of patients in the H_T2DM cohort. The results of the biochemical tests revealed that alanine aminotransferase (ALT) (P < 0.001), aspartate aminotransferase (AST) (*P* = 0.001) and hemoglobin A1 (HbA1c) (*P* = 0.014) levels were significantly greater in the H_T2DM group than in the L_T2DM group. High-density lipoprotein cholesterol (HDL-C) levels were significantly lower in the H_T2DM group than in the L_T2DM group (*P* = 0.010). To exclude potential confounding effects, we performed subgroup analyses based on smoking status using one-way ANOVA. The results revealed no significant differences in HDL-C levels across different smoking categories within either population (*P* = 0.088), confirming that smoking habits do not contribute to the observed HDL-C discrepancy between the two groups. Instead, this difference may be primarily linked to the distinct dietary patterns of high-altitude populations, who predominantly consume animal oil and meat. Epidemiological evidence indicates that such a diet, rich in saturated fat, can impair reverse cholesterol transport and downregulate hepatic HDL synthesis, thereby reducing HDL-C levels and altering lipid metabolism in plateau residents. Later, we will further study the differences in lipid metabolism between the plateau population and the plain population. In addition, there was no significant difference in serum total cholesterol (TC, *p* = 0.205), triglycerides (TG, *p* = 0.13) and blood glucose AUC (*p* = 0.071) between high- and low-altitude groups. Although statistically non-significant, the observed trend suggests that high-altitude T2DM patients may experience a higher overall 24-hour glycemic load. Suggesting that dietary factors related to lipid and antioxidant intake may not substantially contribute to the observed metabolite variations (**Table 1**). Thus, in our study population, the high-altitude environment did not have an effect on lowering blood glucose.

**Table 1:** Clinical characteristics of T2DM between high-altitude and low-altitude.

Characteristic	H_T2DM	L_T2DM	*P*-value
Age, years	51.54 ± 9.14	55.64 ± 6.73	*P*<0.001
Gender			0.083
Male	59	63	
Female	41	37	
Height, cm	168.71 ± 8.35	165.93 ± 7.72	0.676
Weight, kg	73.55 ± 11.75	64.98 ± 9.40	0.040
BMI, kg/m^2^	25.80 ± 3.44	23.57 ± 2.83	0.082
Systolic blood pressure (mm Hg)	120.40 ± 14.60	123.08 ± 14.67	0.733
Diastolic blood pressure (mm Hg)	79.69 ± 9.68	80.87 ± 10.22	0.399
LDL-cholesterol (mmol/l)	2.62 ± 0.79	2.44 ± 0.85	0.408
HDL-cholesterol (mmol/l)	1.15 ± 0.25	1.31 ± 0.72	0.010
Smoking	28%	30%	0.088
ALT, median(range), U/l	29.99 ± 15.95	20.08 ± 11.67	*P*<0.001
AST, median(range), U/l	20.58 ± 11.72	17.40 ± 6.62	0.001
Triacylglycerides (mmol/l)	1.52 ± 0.77	1.62 ± 0.90	0.130
Total cholesterol (mmol/l)	4.47 ± 0.92	4.33 ± 1.08	0.205
HbA1C (%)	10.32 ± 2.55	8.46 ± 2.11	0.014
Fasting glucose (mmol/l)	7.87 ± 2.10	7.00 ± 2.00	0.334
2 h glucose, OGTT (mmol/l)	16.91 ± 3.97	16.32 ± 4.52	0.313
glucose AUC(mmol/L·h)	24.79 ± 5.52	23.33 ± 5.83	0.0714

T2DM, Type 2 diabetes mellitus; LDL, low density lipoprotein; HDL, high density lipoprotein; ALT, alanine aminotransferase; AST, aspartate aminotransferase; HbA1c, hemoglobin A1. Data are mean ± SD. T-test in continuous variables and chi-square test in categorical data were performed as appropriate. Results were considered significant when *P* < 0.05.

Reports have shown that the prevalence of diabetes at high altitudes is lower than that at low altitudes. However, for patients who already have diabetes, H_T2DM patients tended to be younger, have higher HbA1c levels, and even have more severe symptoms of diabetes than L_T2DM patients.

Therefore, we believe that a high-altitude environment affects the metabolic processes of diabetic patients resulting in higher HbA1c levels than in low-altitude diabetic patients. We further analyzed the metabolism of patients with high-altitude diabetes and low-altitude diabetes.

### Metabolic profiles of the different groups

**Figure 2:**
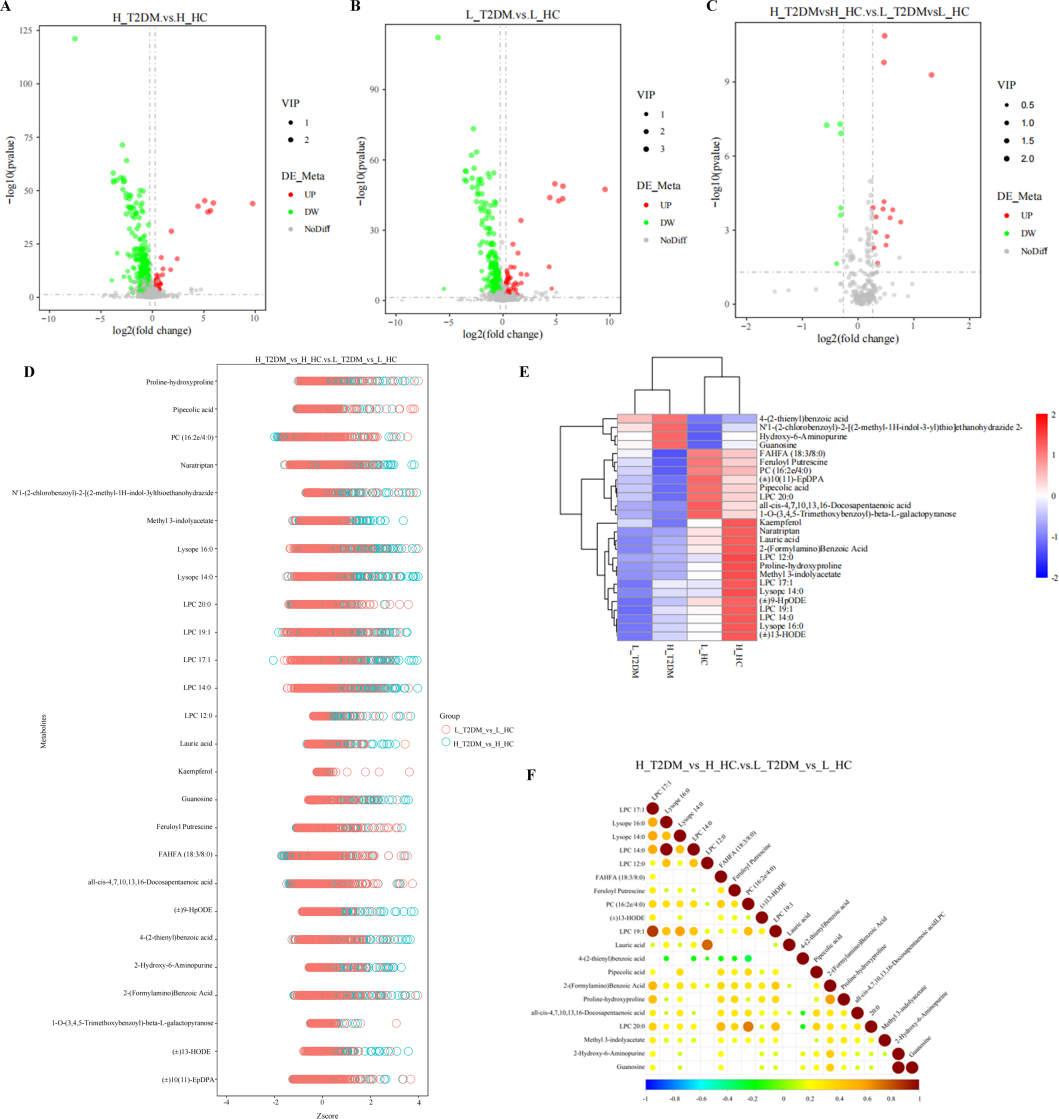
The metabolic profiles of different groups. **(A)** Volcano plots of H_T2DM vs. H_HC. Green dots indicate down-regulated metabolites and red dots indicate up-regulated metabolites **(B)** Volcano plots of L_T2DM vs. L_HC. **(C)** Volcano plots of H_T2DM vs. H_HC vs. L_T2DM vs. L_HC. **(D)** The Z scores of differentially abundant metabolites in both the H_T2DM vs. H_HC and L_T2DM vs. L_HC comparisons. Pink represents the metabolite abundance of people with high altitude diabetes and blue represents the metabolite abundance of people with plains diabetes **(E)** Heatmap of cluster analysis associations in each group. Red represents positive correlation, blue represents negative correlation **(F)** Correlation of differentially abundant metabolite associations with H_T2DM vs. H_HC and L_T2DM vs. L_HC.

We used LC–MS/MS to profile serum samples from 100 H_T2DM, 100 H_HC, 100 L_T2DM, and 100 L_HC individuals using the criteria of FC > 1.2 or <0.833, *P* < 0.05 and VIP > 1. By comparing and analyzing the metabolomes of H_T2DM and H-HC (H_T2DM.vs.H_HC) we obtained differentially abundant metabolites (DMs) related to high-altitude diabetes. The results showed that 412 DMs (32 upregulated and 380 downregulated) related to H_T2DM (**Figure 2A**). This stringent approach ensured that only metabolites showing both statistical significance and biological relevance were selected for downstream analysis. To obtain the DMs related to low-altitude diabetes we subsequently conducted a comparative analysis of the metabolomics between L_T2DM and L_HC (L_T2DM.vs. L_HC, L_HC as a comparison). There were 308 DMs related to L_T2DM, among which 51 DMs were upregulated and 257 DMs were downregulated (**Figure 2B**). To further investigate the metabolic differences between diabetes at high altitudes and low altitudes, we conducted a comparative analysis of 308 DMs associated with L_T2DM and 412 DMs associated with H_T2DM. Finally, 26 DMs were identified, of which 8 DMs were upregulated and 18 DMs were downregulated in L_T2DM (**Table 2**). In other words, there were 26 DMs between diabetic patients at high altitudes and at low altitudes, with 8 playing a downregulated role in diabetic patients at high altitudes and 18 playing an upregulated role (**Figure 2C**).

**Table 2:** The different metabolites of T2DM between high-altitude and low-altitude.

Metabolites	Log2FC	P value	VIP	Up/Down
PC (16:2e/4:0)	-0.31	<0.01	2.20	down
LPC 17:1	0.47	<0.01	2.17	up
LPC 12:0	1.32	<0.01	1.96	up
Lysope 16:0	0.48	<0.01	2.13	up
Lysope 14:0	0.58	<0.01	1.92	up
Feruloyl Putrescine	-0.56	<0.01	2.27	down
FAHFA (18:3/8:0)	-0.32	<0.01	2.23	down
LPC 14:0	0.46	<0.01	2.02	up
LPC 20:0	-0.31	<0.01	1.68	down
(±)13-HODE	0.32	<0.01	1.56	up
Lauric acid	0.46	<0.01	1.28	up
Guanosine	0.77	<0.01	1.22	up
Methyl 3-indolyacetate	0.32	<0.01	1.21	up
N’1-(2-chlorobenzoyl)-2-[(2-methyl-1H-indol-3-yl)thio]ethanohydrazide	0.52	<0.01	1.09	up
all-cis-4,7,10,13,16-Docosapentaenoic acid	-0.30	<0.01	1.65	down
LPC 19:1	0.35	<0.01	1.53	up
2-(Formylamino)Benzoic Acid	0.45	<0.01	1.27	up
(±)9-HpODE	0.50	<0.01	1.10	up
Pipecolic acid	-0.31	<0.01	1.63	down
Proline-hydroxyproline	0.62	<0.01	1.24	up
2-Hydroxy-6-Aminopurine	0.57	<0.01	1.23	up
Naratriptan	0.32	<0.01	1.18	up
(±)10(11)-EpDPA	-0.32	0.01	1.30	down
Kaempferol	0.35	0.02	1.23	up
1-O-(3,4,5-Trimethoxybenzoyl)-beta-L-galactopyranose	-0.38	0.02	1.30	down

To comprehensively investigate the levels and regulatory processes of these 26 DMs we applied Z score transformation (a value based on the relative level of the metabolite) and conducted cluster analysis. The absolute value of the Z score distribution in the L_T2DM group was smaller than in the H_T2DM group, indicating that the levels of these metabolites were generally lower in the L_T2DM group than in the H_T2DM group (**Figure 2D**). The clustering results showed that the levels of 4-(2-thienyl) benzoic acid, N’1-(2-chlorobenzoyl)-2-[(2-methyl-1H-indol-3-yl)thio]ethanohydrazide, 2-hydroxy-6-aminopurine, and guanosine were significantly different between the H_T2DM and L_T2DM groups (**Figure 2E**). In addition, correlation analysis of the levels of the top 20 metabolites revealed that N’1-(2-chlorobenzoyl)-2-[(2-methyl-1H-indol-3-yl)thio]ethanohydrazide and guanosine levels were strongly positively correlated with 2-hydroxy-6-aminopurine levels (**Figure 2F**). These findings suggest that N’1-(2-chlorobenzoyl)-2-[(2-methyl-1H-indol-3-yl)thio]-ethanohydrazide, guanosine and 2-hydroxy-6-aminopurine were closely related to H_T2DM.

### Identifying key metabolites

**Figure 3:**
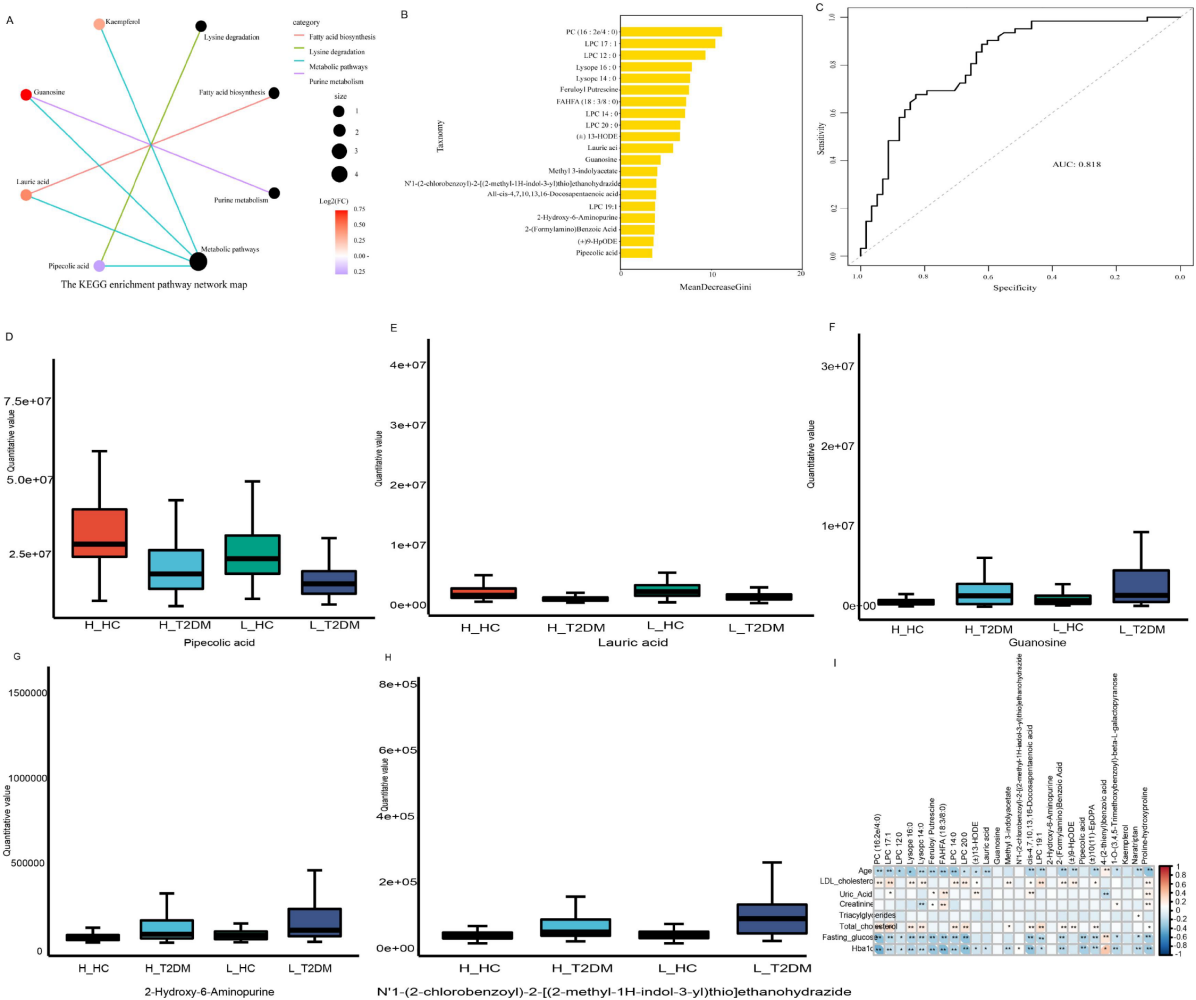
Screening of differentially abundant metabolites. **(A)** KEGG enrichment pathway network map. **(B)** Random forest results. **(C)** Receiver operating characteristic (ROC) curve. **(D)** Levels of N’1-(2-chlorobenzoyl)-2-[(2-methyl-1H-indol-3-yl)thio]-ethanohydrazide. **(E)** Levels of guanosine in each group. **(F)** Levels of 2-hydroxy-6-aminopurine. **(G)** Levels of pipecolic acid in each group. **(H)** Levels of lauric acid in each group. **(I)** Correlation results with biochemical indicators. The closer the AUC is to 1, the more accurate the model prediction.

To further screen the key differentially abundant metabolites between high-altitude and low-altitude diabetic patients, KEGG enrichment analysis(The version is 2/20/17(c)Kanehisa Laboratories) (9)was performed on the 26 DMs. The results showed that the DMs were enriched mainly in the KEGG pathways of lysine degradation, fatty acid biosynthesis, purine metabolism and metabolic pathways, and four metabolites, namely, pipecolic acid, lauric acid, guanosine and kaempferol, were the most prominent (**Figure 3A**). We subsequently conducted a random forest analysis and the results showed that pipecolic acid, lauric acid and guanosine were among the top 20 biomarkers (**Figure 3B**). Moreover, the area under the ROC curve (AUC) was 0.818, suggesting that the random forest model had high predictive accuracy (**Figure 3C**). Combined with the results shown in **Figure 2F**, N’1-(2-chlorobenzoyl)-2-[(2-methyl-1H-indol-3-yl)thio]-ethanohydrazide, guanosine, 2-hydroxy-6-aminopurine, pipecolic acid and lauric acid, these five metabolites are highly correlated with known mechanisms of altitude adaptation, strongly suggesting that altitude environment (rather than diet) is the primary driver of differences between patients with altitude diabetes and those with plains diabetes (10).

We subsequently analyzed the levels of N’1-(2-chlorobenzoyl)-2-[(2-methyl-1H-indol-3-yl)thio]-ethanohydrazide, guano-sine, 2-hydroxy-6-aminopurine, pipecolic acid and lauric acid in each group. The results showed that there were no significant differences in the levels of these five metabolites between healthy individuals at high altitudes and at low altitudes (**Figures 3D-H**). In addition, 2-hydroxy-6-aminopurine and lauric acid did not significantly differ between H_T2DM and L_T2DM. However, the level of N’1-(2-chlorobenzoyl)-2-[(2-methyl-1H-indol-3-yl)thio]-ethanohydrazide in H_T2DM was lower than in L_T2DM. However, the levels of pipecolic acid and guanosine in H_T2DM were both greater than those in L_T2DM.

To further verify the reliability of these biomarkers, we matched the top 20 biomarkers to the mzCloud (https://www.mzcloud.org/) and mzVault databases (https://mytracefinder.com/tag/mzvault/) (the match between the actual detected mass–charge ratio and the mass–charge ratio reported in the database; the highest score was 100 points) and found that only guanosine and pipecolic acid had high matches in the three databases: both match scores were 88 points except for N’1-(2-chlorobenzoyl)-2-[(2-methyl-1H–indol–3-yl)thio]-ethanohydrazide (**Table 3**). This result illustrates that guanosine and pipecolic acid had high predictive accuracy and are the greatest potential determinants of diabetic metabolic differences between high and low altitudes.

**Table 3:** The matching degree of the top 20 metabolites in three database.

Metabolite name	mzCloud	mzVault	MassList
PC (16:2e/4:0)	No results	79.7	full match
LPC 17:1	No results	87.3	No results
LPC 12:0	No results	88.1	No results
Lysope 16:0	No results	No results	full match
Lysopc 14:0	No results	No results	full match
Feruloyl Putrescine	No results	No results	full match
FAHFA (18:3/8:0)	No results	53.4	No results
LPC 14:0	No results	89.4	No results
LPC 20:0	No results	76.8	No results
(±)13-HODE	No results	73	full match
Lauric acid	86.9	No results	full match
Guanosine	88.7	92	full match
Methyl 3-indolyacetate	No results	No results	full match
N’1-(2-chlorobenzoyl)-2-[(2-methyl-1H-indol-3-yl)thio]ethanohydrazide	41	No results	No results
all-cis-4,7,10,13,16-Docosapentaenoic acid	82.4	No results	full match
LPC 19:1	No results	85.5	full match
2-(Formylamino)Benzoic Acid	No results	No results	full match
(±)9-HpODE	88.1	No results	No results
Pipecolic acid	91.2	89.9	full match

### Correlation of different metabolites with diabetes

To verify whether guanosine and pipecolic acid are determinants of diabetes at high and low altitudes we conducted a correlation analysis between metabolites and disease indicators in diabetic patients to identify metabolites that are only affected by altitude differences. The results showed that guanosine levels were not correlated with biochemical indicators (**Figure 3I**). These findings suggest that changes in guanosine levels are not affected by diabetes and are only associated with changes in altitude.

### Multicenter analysis

**Figure 4:**
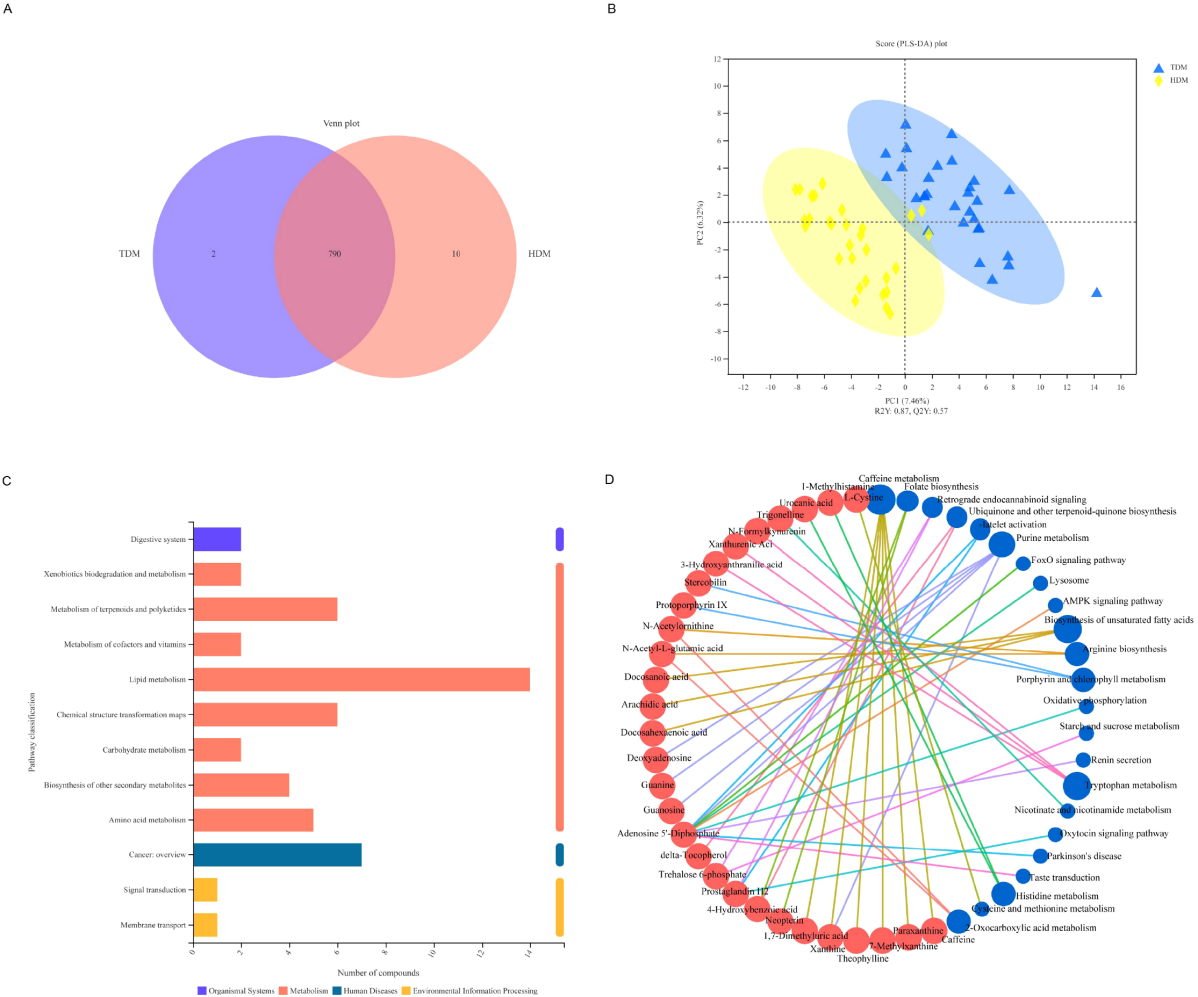
Results of KEGG enrichment pathway and network structure analyses. **(A)** Venn plot for HDM and LDM. **(B)** The PLS-DA score plot and the ranking verification diagram for HDM and LDM (In the scatter plot, the abscissa is the score of the sample on the first principal component, the ordinate is the score of the sample on the second principal component, R2Y represents the interpretation rate of the model, and Q2Y is used to evaluate the prediction of the PLS-DA model. The model is well established when R2Y is greater than Q2Y. For the ranking test, the abscissa represents the correlation between the random group Y and the original group Y, and the ordinate represents the R2 and Q2 scores). **(C)** Pathway classification for HDM and LDM. **(D)** The network structure of metabolites and enrichment pathways.

To confirm the validity of guanosine levels as a potential determinant of metabolic differences in diabetes between high-altitude and low-altitude individuals, we further recruited 30 diabetic patients living at high altitudes (HDMs) and 30 diabetic patients living at low altitudes (LDMs). After application of the inclusion and exclusion criteria, their serum was collected for LC–MS/MS analysis which detected 800 and 792 metabolites in the samples from the HDM and LDM groups, respectively, with 790 metabolites in common (**Figure 4A**). The results of PLS-DA revealed that R2Y was greater than Q2Y, indicating that the model was well established (**Figure 4B**). KEGG enrichment analysis was subsequently performed and showed that the DMs were involved mainly in lipid metabolism (**Figure 4C**). The network structure analysis results revealed that guanosine was still among the top 20 DMs (**Figure 4D**).

Thus, we believe that guanosine is the greatest potential determinant of metabolic differences in diabetic patients between high-altitude and low-altitude individuals.

## Discussion

T2DM is the third most threatening noncommunicable metabolic disease to human health and life safety, following cardiovascular diseases and tumors, and has become a relatively serious worldwide health problem (11). Reports have indicated that the prevalence of diabetes at high altitudes is lower than at low altitudes (12). However, hypoxia at high altitudes has a significant effect on metabolites (13). O’Brien et al. reported that compared with low-altitude individuals healthy individuals residing at high altitudes have increased rates of glycolysis and fat storage mobilization, with decreased isoleucine and glucose levels and increased levels of lactic acid and circulating free fatty acids (palmitic acid, linoleic acid, and oleic acid) (14). Additionally, hypoxia significantly alters hypoxia-inducible factor-1 (HIF-1) DNA binding and the HIF-1α protein, with HIF-1 increasing the supply of glycolytic energy by increasing the expression of enzymes such as pyruvate dehydrogenase kinase (15). Furthermore, hypoxia leads to a decrease in total circulating glutathione levels and an increase in lactic acid and succinic acid levels (16).

In this study, we conducted a comparative analysis of the clinical characteristics and metabolic differences in diabetic patients between high-altitude and low-altitude populations. The results revealed that the weight of patients with T2DM at high altitudes were greater than those at low altitudes, which may be related to oxidative damage caused by the hypoxic environment at high altitudes, which damages the islet cells of the body causing insulin resistance and reducing the utilization rate of glucose. In addition, the characteristics of blood lipids in T2DM patients at high altitudes and low altitudes also differ. HDL levels were relatively low. This may also be related to the fact that people at high altitudes mainly eat animal oil and meat, it may have an impact on the lipid metabolism of the plateau population. Later, we will further study the differences in lipid metabolism between the plateau population and the plain population. In terms of liver function, compared with people in plain areas the serum ALT levels of T2DM patients at high altitudes were greater, indicating that liver damage in diabetic patients at high altitudes was more serious than at low altitudes. Liver damage leads to insulin resistance and an ineffective reduction in blood glucose. In addition, the HbA1c level of T2DM patients at high altitudes was significantly higher than that at low altitudes, which may be related to liver damage affecting insulin resistance and subsequently affecting HbA1c, or it may be caused by insufficient understanding and weak awareness of T2DM and its treatment methods at high altitudes.

To compare the metabolic differences between diabetic patients at high and low altitudes more comprehensively, the metabolic profiles of H_T2DM, H_HC, L_T2DM and L_HC plasma samples were compared. Nontargeted metabolomics based on LC–MS/MS was performed to investigate metabolic changes and screen differentiated metabolites and metabolic pathways. In this cohort study, we found that there were significant differences in the five metabolites of N’1-(2-chlorobenzoyl)-2-[(2-methyl-1H-indol-3-yl)thio]-ethanohydrazide, guanosine, 2-hydroxy-6-aminopurine, pipecolic acid and lauric acid for diabetes between high altitude and low altitude. The DMs were involved mainly in lysine degradation, fatty acid biosynthesis, purine metabolism and metabolic pathways. At high altitudes N’1-(2-chlorobenzoyl)-2-[(2-methyl-1H-indol-3-yl)thio]-ethanohydrazide, guanosine, 2-hydroxy-6-aminopurine, and lauric acid in plasma were upregulated but pipecolic acid was downregulated. A variety of methods combined with analysis revealed that guanosine could be the greatest potential determinant of metabolic differences in diabetes between high-altitude and low-altitude individuals.

Guanosine is a purine nucleoside that is stably released in the brain, and it is a nitrogenous base with relatively low oxidation potential. It is frequently attacked by various active substances and when subjected to oxidative stress is converted into 8-hydroxydeoxyguanosine (8-OHdG) (17). 8-OHdG has been recognized as a biological marker for evaluating the oxidative stress state of the body (18). Numerous studies have shown that 8-OHdG can be used as a potential biomarker of diabetes (19). There are also research reports indicating that the higher the level of 8-OHdG, the more severe the degree of oxidative damage, consistent with our findings (20). The marked elevation of guanosine in high-altitude Tibetans may reflect a dual role of this metabolite in both hypoxia adaptation and glucolipid metabolic regulation.

Emerging evidence suggests guanosine serves as a critical modulator of oxidative stress and HIF-1 signaling—two pathways implicated in the unique diabetic phenotype observed in high-altitude populations. Under chronic hypoxia, guanosine accumulation may act as a compensatory mechanism to mitigate oxidative damage. Guanosine enhances cellular antioxidant capacity by upregulating glutathione synthesis via increased glutamate-cysteine ligase (GCL) activity (21).

Mechanistically, guanosine modulates HIF-1 signaling at multiple levels. Hypoxia-induced guanosine accumulation stabilizes HIF-1α by inhibiting prolyl hydroxylase domain (PHD) enzymes, which are responsible for HIF-1α degradation under normoxic conditions (22). This stabilization promotes the transcription of HIF-1 target genes involved in angiogenesis (VEGF), erythropoiesis (EPO), and glycolysis (GLUT1, LDHA), collectively enhancing oxygen delivery and metabolic adaptation to hypoxia. Notably, our metabolomic analysis reveals that guanosine-mediated HIF-1 activation in high-altitude Tibetans is associated with suppressed fatty acid oxidation and increased glucose utilization, a metabolic shift that may contribute to the lower T2DM prevalence observed in this population compared to low-altitude counterparts.

We acknowledging that chronic hypoxia may influence erythrocyte dynamics (23), our data suggest that the observed guanosine elevation is more likely attributable to systemic metabolic adaptation rather than hematological confounders. This conclusion was supported by Jiayue Gao et.al (24), in their study, it was found that metabolites such as adenosine, guanosine, inosine and xanthine acid were changed under the influence of altitude, and the metabolite content returned to normal after returning from the plateau to the plain, which was not affected by red blood cell lifespan.

In our study, the oxidative damage caused by high-altitude diabetes was more serious than that caused by low-altitude diabetes because of the influence of high-altitude hypoxia, and the levels of guanosine in H_T2DM patients were greater than those in L_T2DM patients. To sum up, guanosine could be a reliable biomarker of metabolic differences in diabetic patients between high altitude and low altitude.

While our cross-sectional design indeed uncertain causal inferences regarding the directionality between high-altitude exposure and diabetic metabolic alterations, we have enhanced biological plausibility through:1)Multiple comparative approaches:Comparisons between healthy vs. diabetic populations within plains or high-altitude(to exclude dietary confounders specific to regions).2)Correlation network analysis: Guanosine levels showed no significant associations with age, LDL cholesterol, triacylglycerides, total cholesterol, creatinine, uric acid, blood glucose, or hemoglobin (**Figure 3I**), indirectly supporting altitude as the primary driver rather than conventional metabolic risk factors. In addition, Yingfeng Gao et.al’s reported that two populations living at different altitudes and found significantly higher levels of both 8-OHdG in the high-altitude group, likely due to hypobaric hypoxia (25).

To sum up, even in a cross-sectional study, our study can provide evidence for guanosine as a key metabolite in high altitude diabetes and plains diabetes.

Besides, we are well aware of other shortcomings. First, as a single-center investigation focused on Tibetans—a population with unique genetic adaptations—the findings may not fully generalize to other high-altitude populations (e.g., Andeans). Second, while we matched immediate environmental factors, lifelong dietary or cultural practices specific to Tibetan communities could contribute to metabolic differences. Thirdly, fasting insulin levels, the homeostasis model assessment of insulin resistance (HOMA-IR), and bioimpedance measurements were not included in our analysis. The lack of these information may mask the potential impact of body composition on glucose regulation in high-altitude environments. In our future multi-center study, we will integrate more comprehensive multi-omics data and various clinical data from different altitude-adapted populations to unravel the influences of genetics, development, and environment.

## Conclusion

Through in-depth comprehensive analysis of serum metabolomics in patients with H_T2DM and L_T2DM, we found that differentially metabolites included mainly pipecolic acid, lauric acid, guanosine and kaempferol, which are involved in lysine degradation, fatty acid biosynthesis, purine metabolism and other metabolic pathways. Machine learning further confirmed that guanosine is the most characteristic biomarker for diabetic patients at high altitudes. This study provides the first evidence of metabolic differences between diabetic patients living in high-altitude and low-altitude areas. In the future, we will carry out multidimensional and large-scale follow-up studies to verify that guanosine is a biomarker of diabetes susceptibility in high-altitude areas.

## Data Availability

The datasets presented in this study can be found in online repositories. The names of the repository/repositories and accession number(s) can be found below: The data of this study have been deposited into the OMIX of China National Center for Bioinformation (CNCB) with accession number PRJCA033459 (OMIX008229), and it is now publicly available as of the date of publication. The access link is https://ngdc.cncb.ac.cn/omix/.
